# Changes of Physiochemical and Enzymatic Activities of *doenjang* Prepared with Different Amount of Rice *koji* during 30 Days of Fermentation

**DOI:** 10.3390/foods10020372

**Published:** 2021-02-09

**Authors:** Yongwoo Jo, Woo-suk Bang, Mina K. Kim

**Affiliations:** 1 Department of Food Science and Human Nutrition and Fermented Food Research Center, Jeonbuk National University, 567 Baekjedaero, Deokjin-gu, Jeonju-si 54896, Korea; jjw9670@naver.com; 2 Department of Food and Nutrition, Yeungnam University, 280, Daehak-ro, Gyeongsan 38541, Korea

**Keywords:** *koji*, fermentation, physiochemical quality characteristics, enzymatic activities

## Abstract

*Koji* is an intermediate fermentation agent, made by inoculating known microorganisms in grains, such as rice, beans, and barley, to hydrolyze starch or protein. The quality of *koji* can influence the final quality of *doenjang*. This study aimed to investigate changes in the physiochemical and enzymatic activities of *doenjang* prepared with different amounts of rice *koji* during a 30-day fermentation period. Three *doenjang* samples were prepared with varying levels of rice *koji*: K1 (11% reduced), K2 (control), K3 (11% increased). Physiochemical characteristics including pH, TA, acid value, moisture content, color, sugar and reducing sugar content, and enzymatic activities including α- and β-Amylase, acidic and neutral protease activities. Samples were taken every 5 days for 30 days of fermentation period. The *doenjang* with a high content of rice *koji* had higher levels of total sugars, reducing sugars, alcohol, and protein enzyme activity than the *doenjang* samples with a lower content of rice *koji* (*p* < 0.05). However, no differences in the physiochemical and enzymatic activities were found between the *doenjang* made with a lower amount of *koji* and the control *doenjang* during fermentation (*p* > 0.05).

## 1. Introduction

*Doenjang* is a traditional soybean fermented food ingredient in Korea. Adding *doenjang* can provide brothy and umami tastes to Korean cuisine due to the presence of amino acids, saccharides (mono-, di-, oligo-, and poly-), and other flavor compounds produced by the fermentation process. In addition to flavor enhancement, *doenjang* is an important source of protein in Korea, where people follow a carbohydrate-rich, cereal-based diet [1]. Along with its high protein content, *doenjang* is rich in essential fatty acids, linolenic acid, and linoleic acid, which can provide essential fatty acids in the Korean diet [2]. In addition to macronutrients, *doenjang* contains bioactive substances, such as aglycone and melanoidine, which are effective in preventing cancer and osteoporosis [3].

*Doenjang* can be manufactured using the traditional method or the commercialized method [4]. Traditional *doenjang* is made by dried soybean brick, called *meju*, and fermentation is heavily dependent on naturally occurring fungi or bacteria. However, in the traditional method, fermentation takes a long time to complete, which limits the ability to mass produce *doenjang* [5]. Recently, companies have developed a method to mass produce commercially-available *doenjang* utilizing *koji*, an intermediate fermentation agent cultured by inoculating *Aspergillus oryzae* in starchy materials such as rice, soybean, barley, and wheat [6]. Koji has widely been used as rapid fermentation of various Asian fermented foods such as miso, *Doenjang*, soy sauce, mirin, sake, and gochujang (red pepper paste) [6]. Manufacturing commercial *doenjang* using *koji* can allow controlling the fermentation process as fermentation agent by direct injection of known bacteria or fungi, primarily *Aspergillus oryzae;* therefore, making standardization and efficient production possible. However, the limitation of commercial *doenjang* is its lack of flavor and low umami taste that is attributed to amino acid degradation of the compounds produced by various microbial communities [4,5,7].

As previously mentioned, the process of making commercial *doenjang* relies heavily on *koji*; therefore, it is necessary to understand *koji’s* role in the *doenjang*-making process. *Koji* contains amylase and proteinase produced by fungi, which converts starch and protein in the raw material into fermentable sugars and amino acids, improving the flavor, taste, and bioactivities of fermented foods [8,9]. Thus, the addition of *koji* can help accelerate the *doenjang* fermentation process. Previous studies related to *koji* have focused on the metabolomics analysis and functional benefits of this enzyme; these have included anti-inflammatory studies [10], anti-obesity studies [11], anti-diabetes studies [12], and studies of volatile components [13].

In Korea, *koji* has traditionally been manufactured using soybean as the main ingredient; however, recently, attempts have been made to produce *koji* using rice, wheat, and barley [14]. In particular, research related to rice *koji* has been conducted on various fermented foods, such as *makgeolli* (Korean traditional alcoholic beverages), *kochujang* (red pepper paste), and *Yakju* (Korean traditional liquor). Most studies on rice *koji* have focused on evaluating the quality of the final products, such as *makgeolli* [15], *Kochujang* [16], and *doenjang* [17,18,19], rather than focusing on rice *koji*. A limited number of studies have focused on *koji* by investigating changes in the selected physical properties and enzyme activities of *koji* made with rice and barley during fermentation and storage [20], the metabolomic profiles of *Bacillus amyloliquefaciens* and/or *Aspergillus oryzae* during rice *koji* fermentation [21]. These studies either focused on the final products made by utilizing *koji*, or the metabolomic profiles of *koji.*

To the best of our knowledge, comprehensive studies have not been conducted to thoroughly determine the relationship between *doenjang* and the quality of the *koji* used for fermentation. While rice *koji* is widely used in Korean liquor manufacturing, it has rarely been applied in the *doenjang*-making process in Korea. Therefore, this study aimed to investigate the changes in the physiochemical and enzymatic activities of *doenjang* prepared with different amounts of rice *koji* during a 30-day fermentation period.

## 2. Materials and Methods

### 2.1. Doenjang Samples Included in This Study

**Figure 1:**
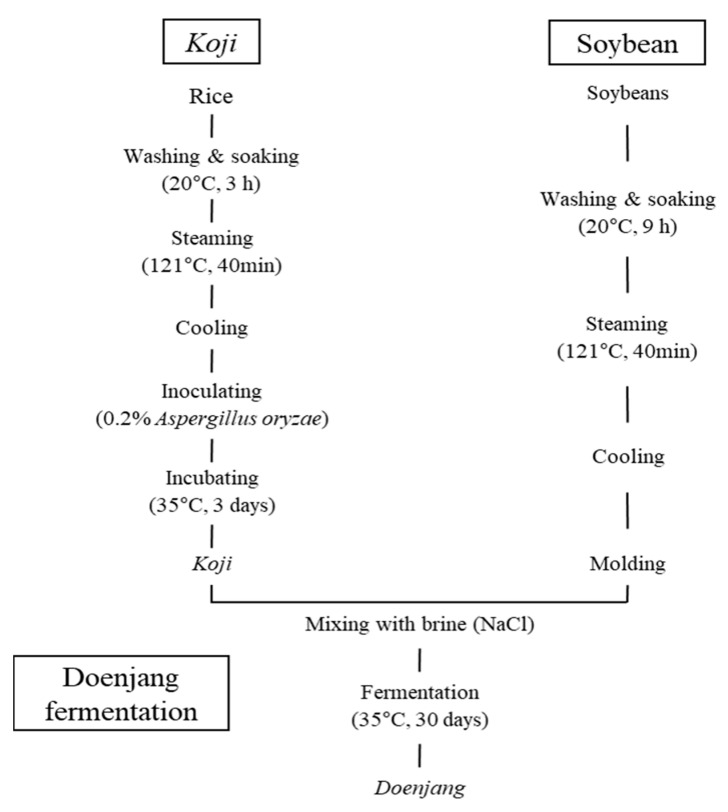
Diagram of *doenjang* sample preparation in this study.

Three *doenjang* samples with different amounts of *koji* were included in this study. The description for each *doenjang* sample is presented in Table 1. All the *doenjang* samples were prepared in the sensory science laboratory in Jeonbuk National University following standard method [5]. Different amounts of *koji* were applied based on weight: 20% (K1), 22.5% (K2), and 25% (K3). K2 had 22.5% *koji* in the *doenjang*, which is standard protocol for the *doenjang*-making process; therefore, it served as the control for this study [5]. The K1 *doenjang* included 20% rice *koji*, which equates about 11.1% *koji* reduction from that in K2, and K3 *doenjang* included 25% rice *koji* in the formulation, which equates about 11.1% increment than control (K2). *Koji* was prepared following a previously published method with a minor modification [5]. A diagram depicting how the *doenjang* samples were prepared is shown in Figure 1.

Briefly, 500 g of rice was washed and soaked in 750 mL of water at a 20 °C for 30 min. Then, water was drained and the soaked rice was sterilize using autoclave (LAC-5060S, Lab Tech, Namyangju, Korea) at 121 °C for 40 min with 0.1 Mpa. Upon autoclave, the rice was immediately cooled to 25 °C. After cooling, the rice was inoculated with *Aspergillus oryzae* (0.2% *w/w*; Yeswine, Boeun-gun, Korea) and incubated for 72 h in an incubator set at 35 °C with 85% humidity. During the incubation, *koji* was mixed thoroughly with sterilized utensils every 24 h. After preparing the *koji*, the *doenjang* samples were prepared using a different amount of *koji*. For *doenjang* preparation, 800 g of soybeans were soaked in 1200 mL of water for 9 h at 20 °C. Then, the water was removed and sterilized at 121 °C for 40 min with 0.1 Mpa using an autoclave. Immediately after autoclave, soybeans were cooled to 25 °C. Then, the cooled soybeans were thoroughly blended with the prepared *koji*, salt, and water using a food processor (NINJA BL682KR, Hai Xin Technology Company, Shenzhen, China). This mixture was then incubated for 30 days at 35 °C with 85% humidity. During incubation, the *doenjang* was mixed evenly using sterilized utensils every 24 h. Samples were taken every 5 days during the 30-day storage period, and the physiochemical characteristics and enzymatic activities were measured.

### 2.2. Physiochemical Analysis of doenjang

Physiochemical characteristics, including pH, moisture content (%), acid value, titratable acidity (%), NH₃-N (%), total sugar and reduced sugar content (mg/g), alcohol content (%), and color (L*, a*, b*) were measured according to the standard methods of analysis listed in the Korean Food Standard Codex by Ministry of Food and Drug Safety. The pH was measured using a pH meter (Lab 850, Schott, Germany) after filtering the 10% (*w/v*) diluted *doenjang* with filter paper (Whatman No. 2; Maidstone, UK). The moisture content of *doenjang* was measured with a moisture analyzer (WBA-110M, Daihan, Korea) using 5 g of *doenjang* paste. The acid value was measured after homogenizing the sample in EtOH/Ether (1:2 *v/v*) solution and titrated with 0.1 N KOH. A 1% phenolphthalein solution was used as an indicator of titration. The titratable acidity (TA) was calculated from the consumption amount by adding 0.1 N NaOH solution to the sample measuring pH and titrating until the pH reached 8.3. Amount of NaOH to neutralize the sample was reported instead of TA. The NH₃-N content was determined by titration with a 0.1 N NaOH solution until the pH was 8.4, then 35% formaldehyde (*v/v*) was added, and re-titration was conducted. Total sugar content was measured by combining 1 mL of the *doenjang* solution with 1 mL of the 5% phenol solution and 5 mL of Conc-H₂SO₄, which was left to stand at room temperature for 30 min. Absorbance at 480 nm was measured (UV-1601, Shimadzu, Tokyo, Japan). The amount of reducing sugar was measured by combining 1 mL of the prepared *doenjang* solution with 1 mL of dinitrosalicylic acid (DNS), which was then mixed at 100 °C for 5 min, and immediately cooled in ice water. Then, absorbance at 550 nm was measured (UV-1601, Shimadzu). A glucose solution was used for the standard curve for the total sugar and reducing sugar content. The glucose-mg per 1 g of each *doenjang* sample (mg/g) were reported. The alcohol content was measured using a filtered 10% *doenjang* solution (*w/v*) with filter paper (Whatman No. 2; Maidstone, UK). A 1 mL *doenjang* and 10 mL of 1 M CrO₄ was thoroughly mixed for 15 min. A-2 mL of distilled water was added to the mixed solution, and absorbance at 600 nm was measured (UV-1601, Shimadzu). Ethanol was used for standard curve. Color was analyzed using a color analyzer (CR-10; Minolta, Osaka, Japan), and lightness (L*), redness (a*), and yellowness (b*) values were recorded. A color standards plate was used with values L* = 94, a* = −0.6, and b* = −3.7. All experiments were analyzed in triplicate.

### 2.3. Enzymatic Activity Analysis of doenjang

The enzymatic activities of *doenjang* were determined based on amylase and protease activities, based on previously published methods [6,7]. To investigate the amylase activity, 10 g of *doenjang* was mixed with 100 mL of distilled water in a shaking water bath (Model Maxturdy-30; Daihan, Seoul, Korea) for 4 h at 25 °C. The mixture was centrifuged at 8000 rpm (Combi-508; Hanil, Gimpo, Korea) for 15 min and filtered (Whatman No.1). For α-amylase, 1 mL of filtered *doenjang* solution was mixed with 2 mL of 1% soluble starch solution (pH 7.0) and left for 30 min at 40 °C. Next, 10 mL of 0.1 N HCl was added to stop the reaction, and then 1 mL of iodine solution was added. Absorbance at 600 nm was measured (UV-1601; Shimadzu, Santa Clara, CA, USA. For β-amylase, 1 mL of filtered *doenjang* solution was mixed with 1 mL of 0.5% soluble starch solution (pH 4.8) and left for 30 min at 30 °C. Next, 1 mL of the DNS solution was added and left for 5 min at 100 °C, then immediately cooled in ice water for a reaction to occur. Absorbance at 550 nm was measured (UV-1601, Shimadzu). The units of enzymatic activity were based on the amount of maltose that occurs for 1 min at 1 mL.

To investigate the protease activity, 3 mL of 0.6% casein solution (pH 3.0 (acidic) and pH 7.0 (neutral)) was added to the *doenjang* solution and preheated for 10 min at 37 °C, and then left to stand for 10 min at 37 °C. Then, 5 mL of 0.4 m trichloroacetic acid was added and left for 20 min at 37 °C. Upon completion of the reaction, the mixture was centrifuged at 3500 rpm for 5 min (Combi-508; Hanil). Then, 2 mL of the supernatant from the mixture was mixed with 5 mL of 0.4 M NaCO₆ and 1 mL of 1 N Folin’s regent and diluted threefold. After 30 min of reaction, absorbance at 660 nm was measured (UV-1601, Shimadzu). The units of enzymatic activity were based on the amount of tyrosine that occurs for 1 min at 1 mL. The enzymatic activity experiments were analyzed in triplicate.

### 2.4. Statistical Analysis

Data analysis was conducted using XLSTAT (v.2020, Addinsoft, Paris, France). One-way analysis of variance was followed by Duncan’s multiple range test to determine differences in the samples at α = 0.05 level.

## 3. Results

### 3.1. Physiochemical Characteristic Analysis

**Figure 2:**
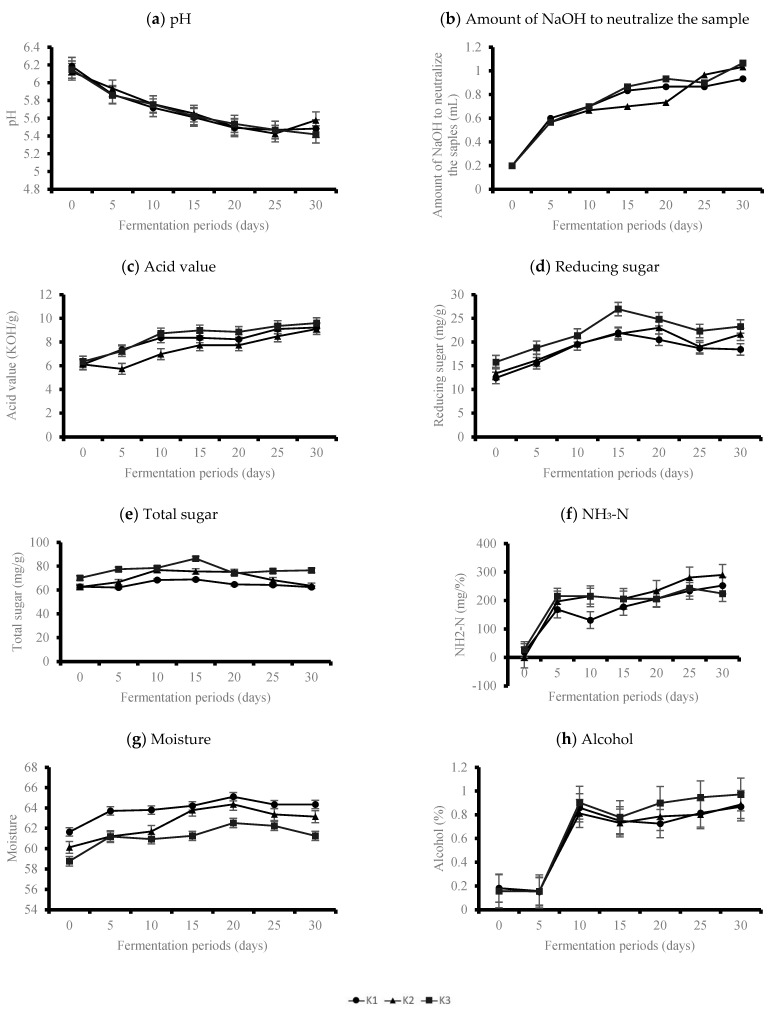
Changes of physiochemical characteristics of *doenjang* during 30 days of fermentation period. (**a**) pH, (**b**) amount of NaOH to neutralize the sample, (**c**) Acid value, (**d**) Reducing sugar, (**e**) Total sugar, (**f**) NH_3_-N (**g**) Moisture, (**h**) Alcohol.

**Figure 3:**
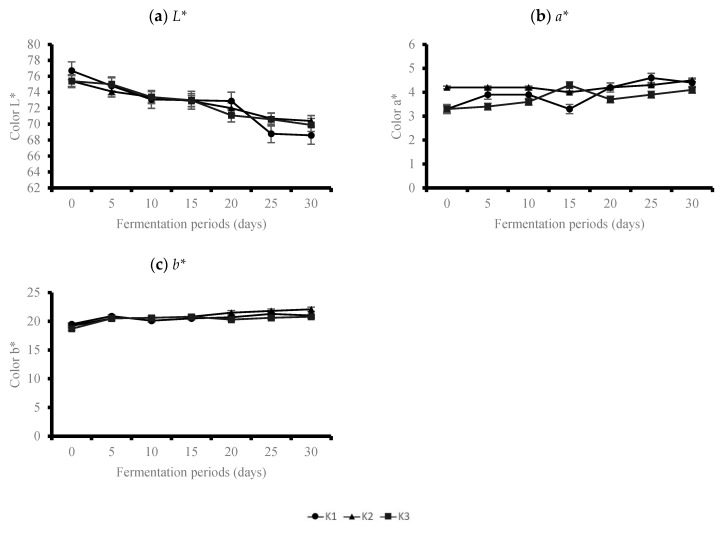
Changes of color of *doenjang* during 30 days of fermentation period. (**a**) L* value (**b**) a* value (**c**) b* value.

**Figure 4:**
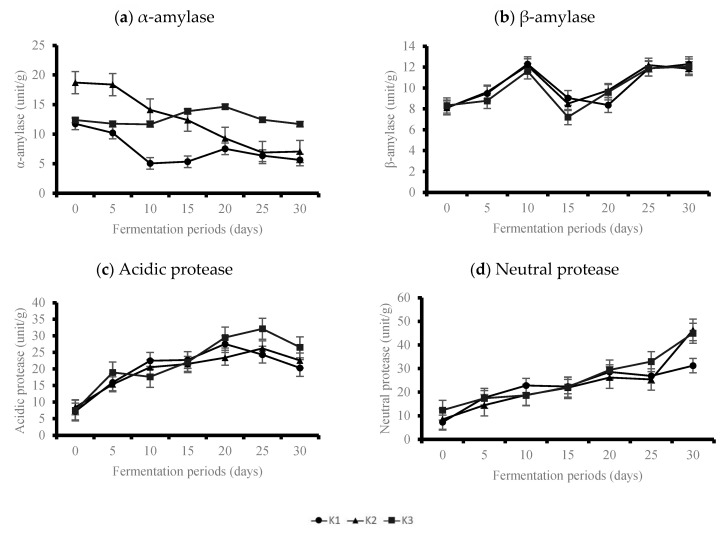
Changes of enzymatic activities of *doenjang* during 30 days of fermentation period. (**a**) α-amylase, (**b**) β-amylase, (**c**) Acidic protease, (**d**) Neutral protease.

The changes in the physiochemical characteristics, color, and enzymatic activities of three *doenjang* samples with different amounts of *koji* (20%, 22.5%, 25%) during a 30-day fermentation period are shown in Figure 2, Figure 3 and Figure 4, respectively Section 3.1.

The pH of the *doenjang* samples decreased as fermentation progressed in all the samples (Figure 2a), and this trend is in agreement with the results reported in a previous study [17]. Briefly, the pH of K1 decreased from 6.15 to 5.47, the pH of K2 decreased from 6.15 to 5.58, and the pH of K3 decreased from 6.15 to 5.41, which is statistically significant (*p* < 0.05). The pH level did not show significant differences between the samples at the beginning of the fermentation process (T0) (*p* > 0.05). However, differences in the pH were observed at T5: K2 had a significantly higher pH (5.93), which was higher than other samples (5.87 for K1 and 5.86 for K3; *p* < 0.05). The pH value of the final fermentation product (T30) was significantly lower in K3 (5.12; *p* < 0.05). K3 had the highest amount of *koji*, which may have influenced its lower pH values. It is estimated that as the amount of *koji* increases, the growth of acid-producing bacteria, such as lactic acid bacteria, becomes active early in the fermentation process, thereby lowering the pH [17].

The amount of NaOH to neutralize the sample of the *doenjang* samples increased as the fermentation progressed in all the samples (Figure 2b). The amount of NaOH to neutralize the sample increased from 0.20 mL to 0.93 mL in K1 from 0.20 mL to 1.03 mL in K2, and from 0.2 mL to 1.07 mL in K3 after 30 days of fermentation (*p* < 0.05). While no significant differences were observed between the samples at T0 (*p* > 0.05), significant differences were observed between the samples from T5 with 0.6 mL for K1, 0.57 mL for K2, and 0.57 mL for K3 (*p* < 0.05). This finding is consistent with the pH results for the samples. The increase in acidity during *doenjang* fermentation is also consistent with the results reported in previous studies [4].

The acid value increased steadily as the fermentation proceeded regardless of the amount of *koji* (Figure 2c). The acid values of K3, which has the highest *koji* content, increased from 6.3 KOH/g to 9.6 KOH/g; the values increased from 6.1 KOH/g to 9.2 KOH/g for K1, and from 6.1 KOH/g to 9.1 KOH/g, for K2; these were statistically significant (*p* < 0.05). The increase in the acid value may have been attributed to the accumulation of the free fatty acids and short-chain organic acids that are formed by the lipase from microorganisms (*Aspergillus oryzae*) [7].

The amount of reducing sugar content increased up to T15 of fermentation, then gradually decreased (Figure 2d). This pattern was observed consistently in all the samples regardless of the amount of *koji* (the level of reducing sugar decreased from 26.96 mg/g to 23.28 mg/g for K3, from 23.02 mg/g to 21.61 mg/g for K2, and from 22.00 mg/g to 18.44 mg/g for K1). The reducing sugar content of K3 was significantly higher at all the time points and tended to increase as the amount of *koji* increased (*p* < 0.05). During *doenjang* fermentation, reducing sugar is produced by the decomposition of the starch material, and the content increases if the production speed is faster than that used for microbial growth. This explains the increase in the amount of reducing sugar until T15 of fermentation [22,23]. Similarly, an increase in the total sugar content was observed up to T15 of fermentation, and then it decreased (Figure 2e). This pattern was observed consistently in all the samples regardless of amount of *koji* included in the *doenjang* fermentation process (86.43 mg/g to 76.47 mg/g for K3, 76.82 mg/g to 63.54 mg/g for K2, and 68.89 mg/g to 62.42 mg/g for K1). The reduction in total sugar content during the fermentation process was as expected because previous studies have reported that sugar can be used as a substrate for microbial growth especially in *doenjang* fermentation [24]. The total sugar content was significantly higher for K3 at all time points (*p* < 0.05). Furthermore, differences between the samples during the fermentation period were statistically significant at all time points (*p* < 0.05).

The content of NH_3_-N was almost non-detectable at T0, but it had a tendency to increase rapidly at T5 and increase continuously thereafter (*p* < 0.05; Figure 2f). For example, NH_3_-N increased from 18.72 to 252.10 for K1, from 0.00 to 290.14 for K2, and from 28.08 to 224.63 for K3 from T0 to T30, and the increases were significant (*p* < 0.05). This trend is consistent with the results reported by Lee and Mok (2010) [22], which reported that NH_3_-N, the main ingredient of soybean paste, continuously increased due to the decomposition of soy protein over the fermentation period, especially early in the fermentation process. Ammonia may seem to produce an unpleasant aroma, but it does not have a negative effect on the quality of soybean paste given that it is less than 400 mg% of the standard in food factories [21]. Furthermore, the difference between NH_3_-N based on the content of the *koji* was not significant (*p* < 0.05).

The average of the moisture content of the samples was 62.6%, which tended to increase regardless of the amount of *koji* (Figure 2g). The moisture content of K1 was at its highest, ranging from 61.6% to 64.3%, followed by K2 (60.1% to 63.1%), and K3 (58.8% to 61.3%) (Figure 2b). Moreover, differences in moisture contents between the samples during the fermentation period were observed at all time points of 30-day fermentation period (*p* < 0.05). In general, the moisture content of *doenjang* is determined by the difference in the moisture of the raw material during the manufacturing process, the change in relative humidity during the aging period, and the degree of decomposition of solids during the aging process. However, this is interpreted as an increase in the amount of free water due to the decomposition of the polymer substances through the enzymatic action secreted by the microorganisms and the production of water through microbial metabolism [25,26].

The levels of ethanol in all the samples were almost non-detectable up to T5 (Figure 2h). However, the levels of ethanol increased significantly after T5, and they were highest at T10 of fermentation. A slight decrease was observed at T15, and then the levels gradually increased again (0.18% to 0.87% for K1, 0.18% to 0.89% for K2, and 0.16% to 0.97% for K3; *p* < 0.05). No significant differences were observed between the three samples up to T20 (*p* > 0.05). However, significantly higher ethanol content was observed in K3, which had the highest koji content (*p* < 0.05). This is because alcohol is produced by the conversion of sugar into alcohol via the fermentation of microorganisms [25]. Therefore, the higher the content of the *koji*, the higher the amount of alcohol.

As a result of physicochemical analysis for 30 days, pH, amount of NaOH to neutralize sample, and acid values showed the same tendency, and reducing sugar and total sugar showed the same pattern.

The color of *doenjang* can have a great influence on consumer preference. The changes in the color of *doenjang* based on to the fermentation period is presented in Figure 3. During 30 days of fermentation, the L* values of the *doenjang* samples (K1-K3) ranged from 75.4 to 76.7 at T0; at T30, the L* value ranged from 70.4 to 68.6, indicating a significant decrease in the L* values in all the samples (*p* < 0.05). Low values of L* upon fermentation may be attributed from the melanoidin formed by the Maillard reaction, which causes the darker color [7,25]. The a* value increased slightly from 3.3 to 4.2 at T0; at T30, the a* value ranged from 4.1 to 4.5 (Figure 3b). The b* value ranged from 18.7 to 19.5 at T0; at T30, the b* value ranged from 20.8 to 22.1 (Figure 3c). A significant increase in the b* values was observed during the 30-day fermentation period, regardless of the amount of *koji* in the *doenjang* (*p* < 0.05).

### 3.2. Enzymatic Analysis Results

The sugar content, which affects the sweetness of *doenjang*, is dependent on the amylase activity produced by the microorganisms involved in fermentation, as well as the protease activity that hydrolyzes the soy protein during fermentation [21,26,27]. Changes in the amylase and protease activity based on the maturation period of *doenjang* prepared by varying the amount of *koji* are presented in Figure 4a–d.

At the beginning of the fermentation process (T0), the α-amylase activity of K2 was 18.7 unit/g, which was significantly higher than that of K1 (11.7 unit/g) and K3 (12.4 unit/g; *p* < 0.05). For K2, the α-amylase activity decreased from 18.7 unit/g to 7.05 unit/g at T30 (*p* < 0.05). However, the α-amylase activity of the other samples was slightly different; it decreased slightly in K1 and K3 up to T10, then it increased up to T20 before decreasing again until T30. Higher α-amylase activity may be due to the large amount of starch in the system, which became the substrate of amylase at the beginning of fermentation. High enzymatic activities continue for as long as the substrate lasts; therefore, the tendency for this activity to decrease was observed in the middle of the fermentation process [18]. β-amylase, a sugar-based enzyme that decomposes starch liquidated by α-amylase, showed a tendency to increase its activity by T10 of fermentation (12.29 unit/g for K1, 12.14 unit/g for K2, and 11.61 unit/g for K3), regardless of the amount of the *koji*, and then it decreased sharply at T15 (9.05 unit/g for K1, 8.50 unit/g for K2, and 7.21 unit/g for K3), before showing a tendency to increase again (*p* < 0.05). The α-amylase activities of the final fermentation product (T30) were significantly higher for K3 than K1 and K2 (*p* < 0.05). However, no significant difference for β-amylase activity was found (*p* > 0.05).

The acidic protease activity increased up to T25 of fermentation, and then it decreased until T30 for all the samples (Figure 4c). The acidic protease activities increased from 7.20 unit/g to 24.33 unit/g for K1, from 8.47 unit/g to 26.24 unit/g for K2, and from 7.48 unit/g to 32.12 unit/g for K3 from T0 to T30, respectively. No significant differences in acidic protease activities between the samples were observed up to T15 (*p* > 0.05); however, differences were observed at T30 (*p* < 0.05). Neutral protease activities of *doenjang* samples increased significantly regardless of the samples (*p* < 0.05); it increased from 7.28 unit/g to 31.12 unit/g for K1, from 8.49 unit/g to 46.40 unit/g for K2, and from 12.36 unit/g to 44.97 unit/g for K3. Similar to the acidic protease activity results, K3 showed significantly higher activity from T25 (*p* < 0.05). This demonstrates that the protease activity increases as the soluble protein or peptide is hydrolyzed to amino acid, which can contribute to the production of NH₃-N by increasing protease activity as the fermentation progresses during the fermentation period [18]. The protease activities of final product (T30), including acidic and neutral, were significantly higher in K3, which had the highest amount of koji (*p* < 0.05).

## 4. Discussion

Amount of *koji* plays an important role in determining the quality of *doenjang*, as starch and/or protein in the raw material is hydrolyzed during fermentation and converted into sugar or amino acids [8,26]. This study focused on how different amounts of *koji* influence the physiochemical characteristics and enzymatic activities of *doenjang*. In this study, the K3 *doenjang* sample, which contained 11% more *koji* than the control (K2), showed increased production of total sugars and reducing sugars due to α-amylase and β-amylase activity and alcohol. High alcohol content in *doenjang* may be the result of the conversion from reducing sugars by yeasts. While the enzymatic activities in K3 increased in comparison to the control (K2), no significant differences were observed in the *doenjang* sample with a lower amount of *koji* (K1) in terms of TA and acid value, NH₃-N, total sugar, and alcohol content. This may be due to the fact that no significant differences were observed in the enzymatic activities, such as α-amylase, β-amylase, and acidic protease, in comparison to the control (K2). Previously, it was reported that the higher the enzymatic activity of *doenjang*, the more by-products produced by fermentation, such as sugars, sugar alcohols, and organic acids, such as succinic, glyceric, fumaric, malic, kojic, citric, and gluconic acids; these results are related to the fermentation rate [28]. This indicates that the differences in physiochemical and enzymatic activities during fermentation were significantly influenced by the amount of *koji*, especially when the amount of *koji* increased. This finding is in agreement with the results reported in previous studies that the amount of total sugar and reducing sugar in *doenjang* showed a tendency to increase significantly based on the content of rice *koji* [29,30].

It is important to note that this study utilized rice *koji*, rather than soybean *koji*, to produce *doenjang*. When comparing the physiochemical characteristics to previously published data on *doenjang* using soybean *koji*, higher enzymatic activity was observed in rice *koji*, especially α-amylase activity, than *doenjang* made with soybean *koji*. It was previously reported that the rate of fermentation is faster when rice *koji* is used than rate of fermentation in *doenjang* using soybean *koji* [1]. The total sugar and reducing sugar content were significantly higher in rice *koji* than soybean *koji* and wheat *koji* [6], and an increase in the total sugar and reducing sugar content was also observed with an increase in the strains (*Aspergillus oryzae*) cultured in rice *koji* [30]. Moreover, the alcohol content and amino nitrogen content was higher in *doenjang* fermented using rice *koji* than when using soybean *koji* [29].

Amylase and protease enzymes were reported to be important factors in determining the sweetness and flavor of *doenjang* [21,24,25]. Previous study reported that rice *koji* has sweet-related aroma characteristics due to the high carbohydrate content of rice and carbohydrate metabolism by amylase activity [6]. Presence of sweet aromatics were previously reported as one of the drivers of liking in *doenjang* among Korean consumers [31]. Thus, the use of rice *koji* in *doenjang* making process may increase the overall acceptability of *doenjang*, however this needs to be further investigated.

## 5. Conclusions

This study investigated the fermentation rate of *doenjang* based on the amount of *koji* content. For *doenjang* with a high *koji* content, many by-products from fermentation are generated and fermentation is fast. In contrast, the *doenjang* with a reduced amount of *koji* did not show a significant difference in the rate of fermentation. These results can be used to help manufacturers determine the amount of *koji* to include when making *doenjang* using commercial methods.

## Figures and Tables

**Table 1:** *Doenjang* with different amount of *koji* samples included in this study.

Sample Code	Bean (g)	Salt (g)	*Koji* (g)	Water (g)
K1	800 (40%)	250 (12.5%)	400 (20%)	550 (27.5%)
K2	800 (40%)	250 (12.5%)	450 (22.5%)	500 (25%)
K3	800 (40%)	250 (12.5%)	500 (25%)	450 (22.5%)

## Data Availability

Data is contained within the article.
